# Zinc ameliorates acrylamide-induced oxidative stress and apoptosis in testicular cells via Nrf2/HO-1/NfkB and Bax/Bcl2 signaling pathway

**DOI:** 10.1080/13510002.2024.2341537

**Published:** 2024-04-17

**Authors:** Ayodeji Johnson Ajibare, Adeyemi Fatai Odetayo, Olabode Oluwadare Akintoye, Luqman Aribidesi Olayaki

**Affiliations:** a Department of Physiology, Faculty of Basic Medical Sciences, College of Medicine, Lead City University, Ibadan, Nigeria; b Physiology Department, Federal University of Health Sciences, Ila-Orangun, Nigeria; c Department of Physiology, Faculty of Basic Medical Science, College of Medicine, Ekiti State University, Ado Ekiti, Nigeria; d Department of Physiology, University of Ilorin, Ilorin, Nigeria

**Keywords:** Acrylamide, apoptotic markers, Bcl-2-like protein 4 (Bax)/B-cell lymphoma 2 (Bcl2), zinc, testicular toxicity, endocrine disruptor, testicular injury, redox imbalance

## Abstract

**Background:**

Acrylamide is a toxic substance formed in some foods that require high-temperature cooking processes and has been implicated as a gonadotoxic agent. Zinc, on the other hand, is a known antioxidant with fertility-enhancing properties. Hence, this study was designed to explore the possible ameliorative effect of zinc in acrylamide-induced gonadotoxicity.

**Methods:**

Twenty-four male Wistar rats were randomized into control, acrylamide (10 mg/kg of acrylamide), acrylamide + 1 mg/kg of zinc, and acrylamide + 3 mg/kg of zinc. The administration was via the oral route and lasted for 56 days.

**Results:**

Zinc treatment ameliorated acrylamide-impaired sperm quality, normal testicular histoarchitecture, and hormonal balance, which was accompanied by increased testicular malondialdehyde and interleukin-1β and decreased testicular superoxide dismutase (SOD) and catalase (CAT). Furthermore, zinc prevented acrylamide-induced downregulation of testicular nuclear factor erythroid 2-related factor 2 (Nrf2), heme oxygenase-1 (HO-1), and B-cell lymphoma 2 (BCl2) expression and upregulation of testicular nuclear factor kappa B (NF-κB) and bcl-2-like protein 4 (bax) expression.

**Conclusion:**

In conclusion, zinc may protect against acrylamide-induced testicular toxicity, mediated by its antioxidant, anti-inflammatory, and antiapoptotic effects.

## 1. Introduction

Infertility has remained a major cause of public health concern, with global statistics at an average of 65 million people affected. Male infertility is also reported to be responsible for about 7 out of every case of infertility [1]. While many of the causes of male infertility seem elusive, there is a growing concern about the role of xenobiotics in the involvement of many previously reported idiopathic male factor causes of infertility [2]. Acrylamide is both a xenobiotic and a ubiquitous hazardous chemical regularly used in various industries and is usually discovered in foods high in carbohydrates that are cooked at high temperatures [3]. The major source of exposure is through food, cigarette smoke, and occupational exposure [3,4]. It is a toxic chemical formed in some foods that require high-temperature cooking processes such as baking, roasting, and frying. Acrylamide is produced from the combination of sugars and amino acids (especially asparagine) naturally found in food [5]. Acrylamide has been linked with several health challenges, such as infertility [6,7].

Cytochrome P450 enzymes convert acrylamide to glycidamide, which is then converted into reactive superoxides. These superoxides can bind with DNA to form adducts, leading to chromosomal abnormalities and mutations [8]. Because acrylamide generates reactive oxygen species (ROS), it can also damage biological macromolecules like lipids, proteins, and DNA, causing oxidative stress. A transcription factor linked to immunity and inflammation, the NF-kB pathway, can also be triggered by acrylamide [9]. Inflammatory cytokines can be produced due to NF-kB activation, including tumor necrosis factor-alpha (TNF-alpha) and interleukin-1 beta (IL-1 Β). Finding treatment targets capable of reducing their effects is even more important, given the intricacy of the biochemical mechanisms underlying acrylamide-induced toxicity [10].

A potential therapeutic target is the Nrf2 pathway. This transcription factor regulates gene expression in antioxidant defense and detoxification via an antioxidant response element (ARE) that activates heme oxygenase-1 (HO-1) [11]. Superoxide dismutase (SOD) and catalase are two antioxidant enzymes produced as a sequel to Nrf2 activation that can scavenge reactive oxygen species (ROS) and protect against oxidative stress when Nrf2 is activated [12]. In addition, Nrf2 can negatively regulate the NF-κB pathway by upregulating IκBα expression, which inhibits the activation of NF-κB. Moreso, accumulating evidence points to the fact that the interplay between the Nrf2/NF-kB pathway tightly regulates both inflammation and oxidative stress and might have a role to play in acrylamide-induced testicular toxicity [13,14]. Furthermore, Nrf2 also prevents apoptosis by regulating Bax/Bcl2 ratio [15].

Zinc, a micronutrient possessing antioxidant properties, has been proposed as a potential mitigating agent for testicular toxins. The optimal development of cells, differentiation, immune function, and male reproductive health necessitates the presence of zinc [16]. Studies have demonstrated that Zinc can enhance the upregulation of Nrf2, leading to a subsequent increase in antioxidant enzymes like CAT and SOD. Furthermore, empirical evidence has shown that zinc sulfate can impede the activation of the NF-κB pathway by augmentation of IBα expression [17].

In light of this, the current study investigated the potential ameliorative effects of Zinc sulfate in the context of AA-induced testicular toxicity via the modulation of the Nrf2/HO-1/NfkB and Bax/Bcl2 pathway. Our findings showed that Zinc exerted a regulatory effect on the Nrf2/HO-1/NfkB and Bax/Bcl2 signaling pathway, resulting in the amelioration of acrylamide-induced testicular toxicity. Additionally, Zinc supplementation enhanced spermatogenesis and restored the testis's altered histoarchitecture and histomorphometric indices in the experimental animals.

## 2. Materials and methods

### 2.1. Chemicals

Acylamide was purchased from May and Baker Ltd. in Dagenham, England. Unless otherwise stated, other chemicals were purchased from Sigma Chemical Company, St Louis, USA.

### 2.2. Animals

Twenty-four male Wistar rats (180–200 g) were bought from the Ekiti State University in Ado-Ekiti, Ekiti, Nigeria. They were taken care of humanely in accordance with the National Institutes of Health's (NIH) criteria for Laboratory Animal Care, and the ARRIVE criteria for reporting experimental results were followed. The Faculty of Basic Medical Sciences, College of Medicine, Ekiti State University's Ethics and Research Committee approved the experimental protocol, which had the protocol number EKSU/P100/2022/11/022, in accordance with the National Research Council's standards for the care and use of laboratory animals.

### 2.3. Experimental protocol

The experimental animals underwent fourteen days of acclimatization and were subsequently randomized into one of the following treatment groups (*n* = 6 per group):

Group 1: Control (received 0.5 ml of Normal Saline)
Group 2: Received Acrylamide (AA) 10 mg/kg b.w
Group 3: Received Acrylamide (AA) + 1 mg/kg/day of elemental zinc
Group 4: Received Acrylamide (AA) + 3 mg/kg/day of elemental zinc.

The dosage of 1 and 3 mg/kg of elemental zinc used in this study are equal to about 11 and 30 mg/day for humans, which are within the recommended dietary intake of 11 mg and the tolerable upper intake level of 40 mg [18]. The dosage of acrylamide used in this study is based on the data on occupational exposure to acrylamide in human [19], and it is similar to the dosage used and reported by Farag et al. [20] and Pourentezari et al. [21].

### 2.4. Sample collection

All treatments lasted 56 days, and overnight fasted animals were sacrificed on the 57th day. The rats were anesthetized using 40 mg/kg of ketamine and 4 mg/kg of xylazine intraperitoneal administration [22] and subsequently sacrificed. A cardiac puncture was performed to collect blood, which was then transferred into non-heparinized tubes. The blood was centrifuged at 3000 rpm for 15 min for biochemical analysis. The epididymis was harvested for sperm analysis, while the right and left testes were collected for biochemical analysis and histology, respectively.

#### 2.4.1. Sperm analysis

The computer-assisted sperm analyzer was used for sperm analysis. Each left caudal epididymis was removed, and many 1 mm-deep incisions were made to release its spermatozoa before putting it in a clean petri dish with 2 ml of regular saline solution. Sperm indices like sperm function tests, oscillation index, sperm kinematics, and sperm velocities were measured.

#### 2.4.2. Preparation of tissue for histology and histomorphometry

The left testis was fixed in enough bouin solution and routinely prepared and embedded with paraffin. Five (5) μm slices were cut and stained with hematoxylin and eosin (H&E) for histology evaluation. The epithelial height, seminiferous tubule diameter, Sertoli cell count, Leydig cell count, and germ cell count were all calculated and estimated using Image J software (version 1.53). The cells were counted, measured, and calculated in a circular view section within a specific square area. Systematic photomicrographs were taken using a computer and an OPTO-Edu industrial camera light microscope.

#### 2.4.3. Biochemical analysis

Hormonal assays were carried out exactly as the manufacturers instructed. The levels of follicle-stimulating hormone (FSH), luteinizing hormone, and testosterone in the blood were determined using the manufacturer's instructions (Bio-Inteco, UK). Serum gonadotropin-releasing hormone (GnRH) was also determined according to the manufacturer's instructions (Melsin, China).

The activities of testicular malondialdehyde (MDA), and total catalase (CAT) and superoxide dismutase (SOD)-like activities were measured using a colorimetric method previously established [23,24].

### 2.5. Polymerase chain reaction for expression of genes

The testes from each group were bisected longitudinally, and the initial half was utilized to transcribe genes following the manufacturer's guidelines. Initially, the isolation of total RNA was performed utilizing the TRIzol Reagent from ThermoFisher Scientific. Subsequently, the RNA was subjected to DNAse I treatment (ThermoFisher Scientific) to eliminate any DNA contaminants. Subsequently, the RNA devoid of DNA was transcribed into complementary DNA (cDNA) through the use of the ProtoScript® First Strand cDNA Synthesis Kit (NEB). The polymerase chain reaction amplification was performed using OneTaq® 2X Master Mix (NEB) (15).

**Table:** 

GENE	FORWARD PRIMER	REVERSE PRIMER	ACCESSION NO
NRF2	GTCAGCTACTCCCAGGTTGC	CAGGGCAAGCGACTGAAATG	NM_001399173
HMOX-1	CGACAGCATGTCCCAGGATT	AGGAGGCCATCACCAGCTTA	NM_012580
NFKB	TTCAACATGGCAGACGACGA	AGGTATGGGCCATCTGTTGAC	NM_001276711
BCL 2	GCGTCAACAGGGAGATGTCA	TTCCACAAAGGCATCCCAGC	NM_016993
BAX	AAACTGGTGCTCAAGGCCC	GGGTCCCGAAGTAGGAAAGG	NM_017059

#### 2.5.1. Statistical analysis

The bar charts depict mean ± SD values (*n* = 6) of the normalized gene expression ratios calculated from gel electrophoresis band densitometry using ImageJ software. The gene expression ratios were normalized to the β-Actin control. The gel images show representative results for each experimental group. The test for normalcy was performed using the ‘Kolmogorov–Smirnov Test of Normality’, and one-way ANOVA followed by post-hoc Tukey tests were conducted using GraphPad Prism 9 to determine statistical significance between groups (*p* ≤ 0.05).

## 3. Results

All markers of sperm function, such as sperm count, normal morphology, vitality, and overall motility, when contrasted with the control group, were significantly deranged on Acrylamide exposure (Table 1). Table 2 also showcased a similar trend wherein acrylamide exposure significantly deranged sperm kinematic parameters such as VAP, VSL, VCL, ALH, BCF, linearity, straightness, and wobble. There was significant dose-dependent mitigation of these acrylamide-induced derangements. Remarkably, while the higher dose of zinc exhibited a more pronounced capacity to counteract acrylamide's impact on sperm function parameters than the lower dose, both lower and higher doses of zinc displayed significant mitigation of the substantial reductions in sperm count, morphology, vitality, motility, and kinematic parameters induced by acrylamide.

**Table 1.** Ameliorative effect of zinc on acrylamide-impaired sperm parameters.

	CONTROL	AA	AA + LZN	AA + HZN
SPERM COUNT	43.57 ± 15.41	14.92 ± 1.46*	38.3 ± 11.14^*#^	45.30 ± 8.06^#$^
MORPHOLOGY	100.0 ± 0.00	25.83 ± 10.83*	92.08 ± 5.22^*#^	99.08 ± 4.04^#$^
VITALITY	100.0 ± 0.00	55.00 ± 7.30*	100.0 ± 0.00^#^	100.0 ± 0.00^#^
TOTAL MOTILITY	96.67 ± 2.11	56.33 ± 3.70	76.62 ± 4.30^*#^	83.67 ± 4.29^#$^

Data were analyzed using one-way analysis of variance (ANOVA), which was then followed by Tukey's multiple post hoc test and level of significance was determined at *p* < 0.05. CON, AA, LZ, and HZ are referred to as control, acrylamide, zinc low dose, zinc high dose respectively. The symbols *, #, and $ indicate the levels of statistical significance compared to CONTROL, AA, AA + LZN respectively.

**Table 2.** Ameliorative effect of zinc on acrylamide-impaired sperm motility

	CONTROL	AA	AA + LZN	AA + HZN
VAP	9.917 ± 1.36	4.167 ± 1.38*	8.467 ± 0.24^*#^	10.12 ± 1.19^#$^
VSL	13.12 ± 1.54	4.950 ± 1.60*	10.93 ± 0.47^*#^	12.87 ± 1.34^#$^
VCL	21.93 ± 1.52	13.58 ± 1.25*	20.98 ± 1.16^*#^	21.92 ± 1.51^#^
ALH	1.297 ± 0.20	0.4333 ±0.20*	1.300 ± 0.08^#^	1.300 ± 0.09
BCF(HZ)	1.658 ± 0.10	0.8667 ± 0.29*	1.283 ± 0.07^*#^	1.327 ± 0.12^*#^
LINEARITY	0.7033 ± 0.07	0.3217 ±0.14*	0.5250 ± 0.03^*#^	0.5100 ± 0.07^*#^
STRAIGHTNESS	1.810 ± 0.39	0.6883 ± 0.33*	1.302 ± 0.084^*#^	1.8683 ± 0.13^#$^
WOBBLE	0.4867 ± 0.04	0.3050 ± 0.10*	0.4800 ± 0.03	0.5800 ± 0.09^*#$^

Data were analyzed using one-way analysis of variance (ANOVA), which was then followed by Tukey's multiple post hoc test and level of significance was determined at *p* < 0.05. CON, AA, LZ, and HZ are referred to as control, acrylamide, zinc low dose, zinc high dose respectively. The symbols *, #, and $ indicate the levels of statistical significance compared to CONTROL, AA, AA + LZN respectively.

**Figure 1.**
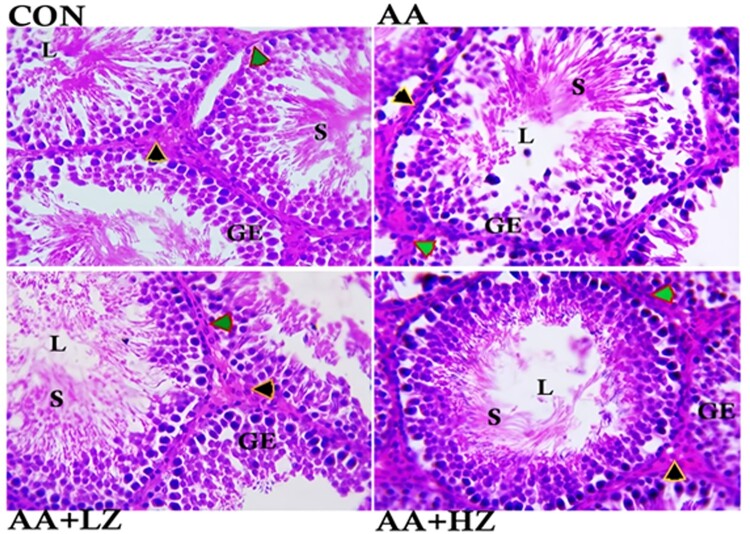
Photomicrograph showing the histoarchitecture changes, Sertoli cell count, Leydig cell count, Germ cell count, Diameter of the seminiferous tubules, Luminal diameter, and Epithelial height of the testis. Control group revealed normal arrangement of the seminiferous tubules epithelium, clusters of sperm cells at the lumen, statistical significant increase in germ cell and Sertoli cell (**p < 0.001), and statistically significant increase in the number of Leydig cells (*p < 0.001). AA group revealed severe degeneration of seminiferous tubules epithelium, loss of sperm cells at the lumen, and degeneration of the interstitial cells. Significant decrease in germ cell, Sertoli cell and Leydig cell count (*p < 0.001) when compared with the control group. There was also a significant increase in the luminal diameter and epithelial height when compared to other group (*p < 0.001). AA + LZ group revealed moderate degeneration of seminiferous tubules epithelium, loss of sperm cells at the lumen and statistically significant increase in the Leydig cells, Sertoli cells and germ cells count when compared with the AA group (*p < 0.001, **p < 0.01). There was also a significant reduction in luminal diameter and epithelial height when compared with AA group (*p < 0.001, **p < 0.01). AA + HZ group revealed moderate loss of seminiferous tubules epithelium, statistical increase in interstitial cells, germ cell, and Sertoli cell when compared with AA group (**p < 0.001), and a significant reduction in the luminal diameter when compared with AA group. (Black arrow: Leydig cells, Green arrow: myoid cells, GE: Germinal epithelium, L: Lumen, S: Spermatids.) Photomicrograph at x800 magnification, using H&E staining.

Furthermore, exposure to acrylamide induced notable structural aberrations in the testes. This was evident in the pronounced degeneration of the epithelium lining the seminiferous tubules, the loss of sperm cells, and the degeneration of interstitial cells. These deviations were contrasted with the normative control group, as illustrated in Figure 1. Within the low-dose group, a significant decline in the integrity of the seminiferous tubule lining and a reduction in the number of sperm cells housed within the tubule were evident. Conversely, the high-dose group exhibited a moderate decrease in seminiferous tubule lining integrity.

**Figure 2.**
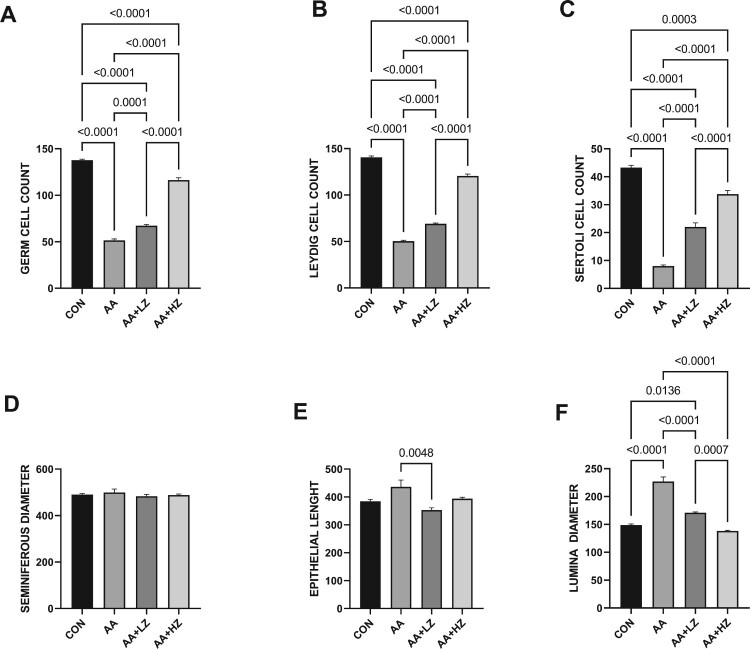
Effect of AA on (A) germ cell count (B) Leydig cell count (C) Sertoli Cell count (D) seminiferous diameter (E) epithelial height (F) luminal diameter. Data were analyzed using one-way analysis of variance (ANOVA), which was then followed by Tukey's multiple post hoc test and level of significance was determined at *p* < 0.05. CON, AA, LZ, and HZ are referred to as control, acrylamide, zinc low dose, zinc high dose respectively.

The presence of acrylamide led to a substantial reduction in the counts of germ, Leydig, and Sertoli cells compared to the control group. Nevertheless, the deleterious impact was alleviated upon introducing zinc, with the most profound ameliorative effects manifesting in animals subjected to a high dosage of zinc (as depicted in Figure 2). Notably, acrylamide did not significantly influence the size of the seminiferous diameter. However, it did engender a marked increase in both the epithelial cells’ height and the luminal space's diameter when contrasted with the control group. These observed alterations were effectively rectified after administering both low and high doses of zinc. However, the beneficial effects were particularly conspicuous in animals treated with higher doses of zinc.

**Figure 3.**
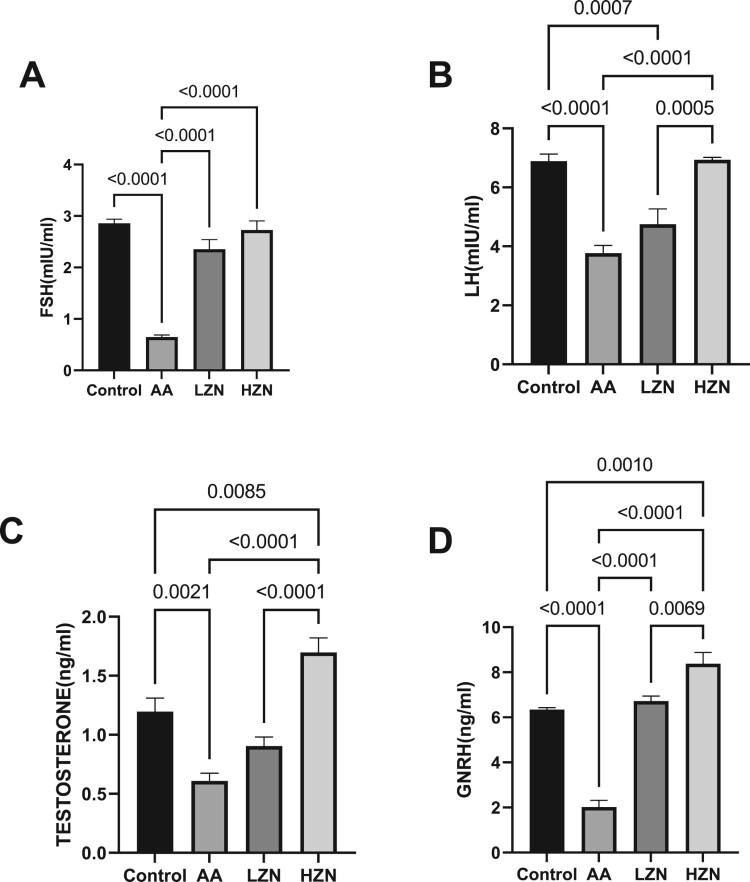
Effect of AA on (A) FSH (B) LH (C) testosterone (D) GnRH. Data were analyzed using one-way analysis of variance (ANOVA), which was then followed by Tukey's multiple post hoc test and level of significance was determined at *p* < 0.05. CON, AA, LZ, and HZ are referred to as control, acrylamide, zinc low dose, zinc high dose respectively

In addition, acrylamide significantly decreased serum FSH, LH, and testosterone compared with the control group. The observed hormonal imbalance was abrogated by zinc treatment. The animals treated with high doses of zinc exhibited a better ameliorative effect than their counterparts treated with low doses (Figure 3).

**Figure 4.**
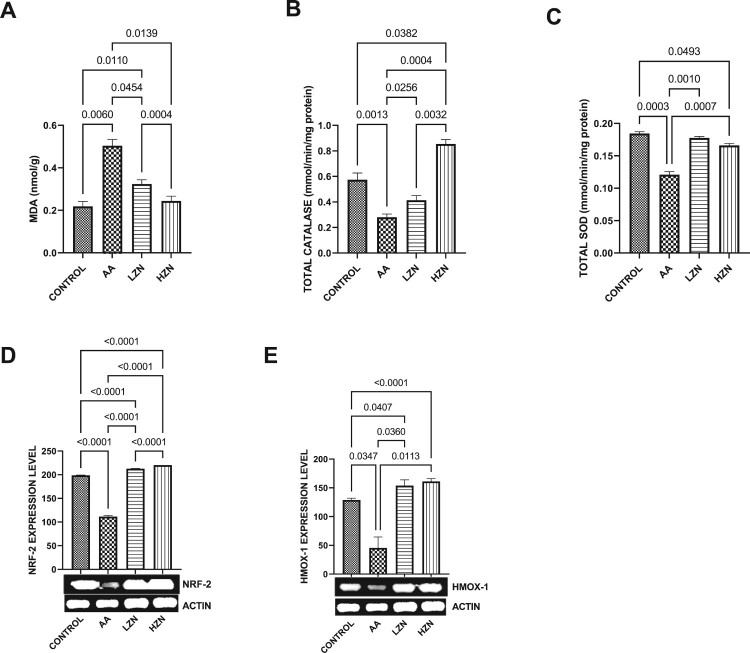
Effect of AA on testicular (A) MDA (B) total catalase-like activities (C) total SOD-like activities (D) NRF-2 (E) HMO-1. The values for the quantified bands of the specified genes from each sample in the five groups were expressed as means ± SEM, where *n* = 6. Data were analyzed using one-way analysis of variance (ANOVA), which was then followed by Tukey's multiple post hoc test and level of significance was determined at *p* < 0.05. CON, AA, LZ, and HZ are referred to as control, acrylamide, zinc low dose, zinc high dose respectively

**Figure 5.**
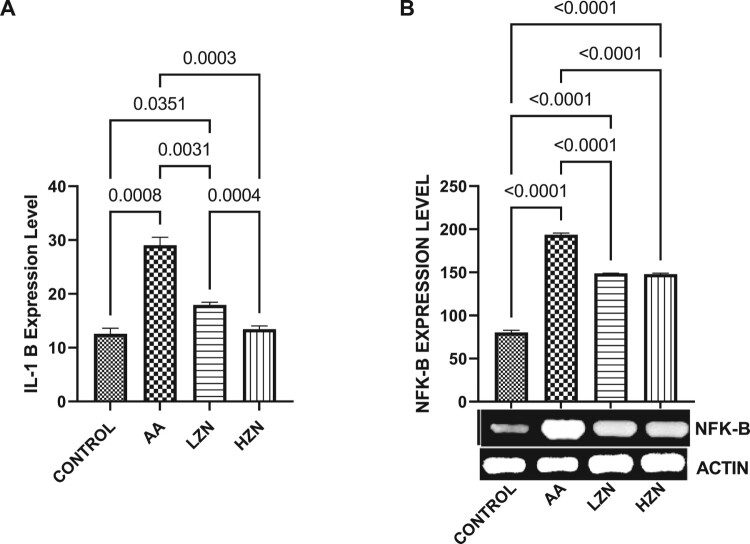
Effect of AA on testicular (A) IL-1B (B) NFK-B. The values for the quantified bands of the specified genes from each sample in the five groups were expressed as means ± SEM, where *n* = 6. Data were analyzed using one-way analysis of variance (ANOVA), which was then followed by Tukey's multiple post hoc test and level of significance was determined at *p* < 0.05. CON, AA, LZ, and HZ are referred to as control, acrylamide, zinc low dose, zinc high dose respectively.

Acrylamide significantly increased testicular MDA and decreased testicular SOD-like, CAT-like, NRF2, and HMOX-1 activities compared with the control (Figure 4). These observed alterations were ameliorated by Zn treatment, although animals treated with high dose exhibited better ameliorative effects than their counterparts treated with the low dose. In Figure 5, there was a significant increase in the testicular IL-1B and NFKB upon acrylamide exposure. This increase in inflammatory markers was significantly reverted with zinc treatment in a dose-dependent fashion, with the high dose being more effective.

**Figure 6.**
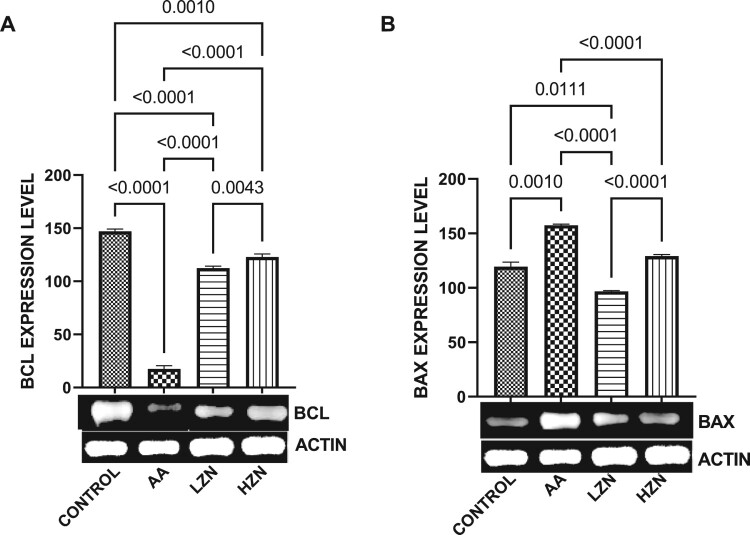
Effect of AA on the testicular expression of (A) BCL2 (B) BAX. The values for the quantified bands of the specified genes from each sample in the five groups were expressed as means ± SEM, where *n* = 6. Data were analyzed using one-way analysis of variance (ANOVA), which was then followed by Tukey's multiple post hoc test and level of significance was determined at *p* < 0.05. CON, AA, LZ, and HZ are referred to as control, acrylamide, zinc low dose, zinc high dose respectively.

Furthermore, in Figure 6, acrylamide exposure resulted in apoptosis within the testicular cells evidenced by a significant decrease in testicular BCL2 and a concomitant increase in Bax expression. These observed alterations were reverted by zinc administration, although the animals treated with higher doses exhibited better ameliorative effect.

## 4. Discussion

The testis, the major organ responsible for steroidogenesis and spermatogenesis in males, is one of the major targets for acrylamide [25,26]. In this study, acrylamide impaired testosterone synthesis (steroidogenesis) and sperm quality in male rats. Rats exposed to acrylamide showed a significant decrease in sperm quality, evident by a significant reduction in sperm count, normal morphology, vitality, and total motility. It also decreased VAP, VSL, VCL, ALH, BCF, linearity, straightness, and wobble. This finding is similar to the findings of Kermani-Alghoraishi et al. [27] and Zhang et al. [28]. The decreased testosterone synthesis and sperm quality following acrylamide exposure could result from acrylamide-induced testicular histopathological findings from this study. Testosterone is primarily synthesized in the Leydig cells, while sperm is produced in the Sertoli cells of the testis. The findings from this study that acrylamide significantly decreased testicular germ cells and Leydig and Sertoli cells indicate that acrylamide impaired testicular functions by directly destroying testicular cells. Aside from the direct toxic effect of acrylamide on the testis, acrylamide also impaired the hypothalamic-pituitary-testicular (HPT) axis [29]. The testis is under the regulation of the HPT axis. The hypothalamus synthesizes GnRH, which stimulates the production and release of FSH and LH. The released FSH regulates spermatogenesis, while LH stimulates testosterone synthesis (steroidogenesis) and spermatogenesis. The observed decrease in GnRH, FSH, LH, and testosterone indicated that acrylamide not only impairs testicular functions by damaging the testicular cells but also distorts the HPT axis and causes hormonal imbalance. The findings that acrylamide impaired the HPT axis are similar to that of Gül et al. [30] and Erdemli et al. [31]. These observed impairment in testicular functions can be ascribed to oxidative stress [32,33], inflammatory response [23,34], and apoptosis [35,36].

The findings from this study that acrylamide exposure led to an increase in oxidative stress and inflammatory response agreed with our speculation about the observed decrease in testicular functions. Acrylamide exposure led to an oxido-inflammatory response by significantly increasing testicular MDA and IL-1β and decreasing testicular catalase and SOD. These findings agreed with the study of Kopańska et al. [37]. The acrylamide-induced oxido-inflammatory response could be associated with its effect on the Nrf2/HO-1/NfkB signaling.

Nrf2 is a cytoprotective and a key transcription factor regulating numerous cell homeostasis by responding to oxidative and toxic insults [11]. Mainly, Nrf2 regulates the basal and induced phase II antioxidant proteins transcription responsible for removing reactive oxygen species (ROS), thereby protecting against the accumulation of toxic metabolites that reduce the antioxidant status [14]. Nrf2 regulates the expression of different cytoprotective genes, such as the ones that regulate the synthesis of glutathione and HO-1 (potent antioxidants). Apart from being an antioxidant, HO-1 also regulates different important biological activities such as inflammation, apoptosis, cell proliferation, and angiogenesis [11]. NFκB, on the other hand, is a major regulator of inflammatory responses. Its overexpression is a hallmark of chronic inflammatory diseases [38] since it stimulates the release of pro-inflammatory markers such as IL-1β [39]. Nrf2 inhibits the stimulation of NFκB signaling by activating HO-1 expression, which improves the antioxidant status and, in turn, inhibits ROS-mediated activation of NFκB [40]. Interestingly, overexpression of NFκB inhibits the transcriptional activity of Nrf2, thereby leading to oxidative stress [41]. Hence, the communication, or ‘crosstalk,’ between Nrf2/HO-1 and NFκB signaling is important for protecting cells from oxidative stress and inflammation.

Furthermore, apoptosis is a major outcome of Nrf2 inhibition by downregulating the activity of antiapoptotic-related BCl2 and upregulating proapoptotic-related Bax [42]. Excessive apoptosis is a major contributing factor to reproductive dysfunction [43]. The results from this study showed that acrylamide induced testicular toxicity through the upregulation of Bax while the antiapoptotic protein Bcl2 was decreased.

This study demonstrated that zinc might potentially attenuate acrylamide-induced testicular toxicity, and Nrf2/HO-1/NfkB and Bax/Bcl2 signaling pathways may be the target for the gonads-protective strategy of zinc. These results agreed with the study of Ganju and Eastman [44], Hamed et al. [18], and Akhigbe et al. [45], that reported that zinc suppressed oxidative stress, inflammation, and apoptosis through the modulation of pro-oxidants, antioxidants, pro-inflammatory, apoptotic and antiapoptotic proteins. The gonado-protective effect of zinc is accompanied by the restoration of testicular histoarchitecture and function by preventing degeneration of seminiferous tubules epithelium, loss of sperm cells at the lumen, degeneration of the interstitial cells and normalization of sperm qualities and reproductive hormones.

## 5. Conclusion

In conclusion, this study shows that acrylamide-induced testicular dysfunction is associated with the modulation of Nrf2/HO-1/NfkB and Bax/Bcl2 signaling. This is coupled with hormonal imbalance and impaired spermatogenesis. However, zinc confers testicular protection by preventing acrylamide-induced Nrf2/HO-1/NfkB and Bax/Bcl2 signaling dysfunction, thus preserving testicular functions. This revealed that zinc might be a potential therapeutic candidate in preventing acrylamide-induced testicular dysfunction.

## Data Availability

The materials for the manuscript, including any relevant raw data, will be freely made available by the corresponding author (Odetayo Adeyemi Fatai) to any researcher who needs them for non-commercial study while protecting participant confidentiality.
